# Individual and family environmental correlates of television and computer time in 10- to 12-year-old European children: the ENERGY-project

**DOI:** 10.1186/s12889-015-2276-2

**Published:** 2015-09-18

**Authors:** Maïté Verloigne, Wendy Van Lippevelde, Elling Bere, Yannis Manios, Éva Kovács, Monika Grillenberger, Lea Maes, Johannes Brug, Ilse De Bourdeaudhuij

**Affiliations:** Department of Movement and Sport Sciences, Ghent University, Ghent, Belgium; Department of Public Health, Ghent University, Ghent, Belgium; Department of Public Health, Sport and Nutrition, University of Agder, Kristiansand, Norway; Department of Nutrition and Dietetics, School of Health Science & Education, Harokopio University, Athens, Greece; Department of Paediatrics, University of Pécs, Pécs, Hungary; Max Rubner-Institut, Federal Research Institute of Nutrition and Food, Karlsruhe, Germany; Department of Epidemiology and Biostatistics and the EMGO Institute for Health & Care Research, VU University Medical Center, Amsterdam, The Netherlands

**Keywords:** Screen time, Children, Parents

## Abstract

**Background:**

The aim was to investigate which individual and family environmental factors are related to television and computer time separately in 10- to-12-year-old children within and across five European countries (Belgium, Germany, Greece, Hungary, Norway).

**Methods:**

Data were used from the ENERGY-project. Children and one of their parents completed a questionnaire, including questions on screen time behaviours and related individual and family environmental factors. Family environmental factors included social, political, economic and physical environmental factors. Complete data were obtained from 2022 child–parent dyads (53.8 % girls, mean child age 11.2 ± 0.8 years; mean parental age 40.5 ± 5.1 years). To examine the association between individual and family environmental factors (i.e. independent variables) and television/computer time (i.e. dependent variables) in each country, multilevel regression analyses were performed using MLwiN 2.22, adjusting for children’s sex and age.

**Results and discussion:**

In all countries, children reported more television and/or computer time, if children and their parents thought that the maximum recommended level for watching television and/or using the computer was higher and if children had a higher preference for television watching and/or computer use and a lower self-efficacy to control television watching and/or computer use. Most physical and economic environmental variables were not significantly associated with television or computer time. Slightly more individual factors were related to children’s computer time and more parental social environmental factors to children’s television time. We also found different correlates across countries: parental co-participation in television watching was significantly positively associated with children’s television time in all countries, except for Greece. A higher level of parental television and computer time was only associated with a higher level of children’s television and computer time in Hungary. Having rules regarding children’s television time was related to less television time in all countries, except for Belgium and Norway.

**Conclusions:**

Most evidence was found for an association between screen time and individual and parental social environmental factors, which means that future interventions aiming to reduce screen time should focus on children’s individual beliefs and habits as well parental social factors. As we identified some different correlates for television and computer time and across countries, cross-European interventions could make small adaptations per specific screen time activity and lay different emphases per country.

**Electronic supplementary material:**

The online version of this article (doi:10.1186/s12889-015-2276-2) contains supplementary material, which is available to authorized users.

## Background

Screen time behaviours (i.e. television and computer time) are the most prevalent sedentary activities among children [1, 2]. Although it is recommended to limit screen time to no more than 2 h per day [3], recent evidence shows that 10- to 12-year-old European boys and girls spend on average 3.5 and 3 h per day, respectively, on screen time behaviours [4]. Various studies have shown an association between screen time behaviour and health risk factors in children, such as overweight and obesity [5–8], suggesting that health promoting interventions should focus on reducing children’s screen time behaviour to improve children’s health.

An important step towards the development of such interventions is to identify correlates and potential determinants of screen time behaviour. Exploring these correlates and possible determinants should be based on a theoretical framework of multi-level determinants. Socio-ecological models [9–11] posit that both individual and environmental level factors are associated with a person’s health behaviour. This suggests that an intervention aiming to improve a person’s health behaviour is more likely to work if it focuses on changing both individual and environmental factors [11]. Specifically for children, the family environment is important, because parents have a major influence on their children’s behaviour and because most screen time behaviour takes place at home [12]. Previous literature review studies have summarized the available evidence regarding the relationship between individual and/or environmental factors and screen time behaviour in children. In general, most evidence was found for a positive association with parental screen time behaviour [12, 13], a home environment with more access to televisions/computers [12, 14] including a television in the child’s bedroom [13, 14], and an inverse association with parental rules regarding screen time behaviour, which means that more parental rules about screen time behaviour is related to less screen time behaviour in children [12]. However, for most investigated factors (including individual variables such as attitude and self-efficacy), the evidence was mixed and inconsistent [12–14]. One possible explanation for the mixed findings is that the definitions of screen time behaviour ranged across studies: some studies investigated total screen time behaviour and others merely television time [12]. However, factors associated with television time may differ from factors associated with computer time.

A previous study of Babey and colleagues [15] among more than 4000 12- to 17-year-old US adolescents has specifically examined correlates of television viewing and computer use separately to determine differences in correlates of the two screen time behaviours. The authors found differences according to the specific screen time behaviour: for example, a higher household income was associated with more television viewing, but with less computer use. Also, boys spent more time watching television than girls, but no sex differences were found for computer use. However, the investigated correlates in this study of Babey and colleagues [15] were at the individual, household and the neighbourhood level, which implies that the majority of those items (e.g. household income, adolescent’ sex, neighbourhood racial composition,…) could be classified as non-modifiable factors. Investigating non-modifiable correlates is useful to identify high-risk subgroups, however, investigating modifiable correlates is needed to inform future interventions on which factors they should focus in order to change the health behaviour. It is therefore important to also investigate a large range of potentially modifiable factors of television and computer time separately.

Furthermore, not only television and computer time can have different correlates, important correlates may also differ between countries or regions. A study of van Sluijs and colleagues [16] examined behavioural correlates (e.g. television viewing after school) as well as parental correlates (e.g. parental BMI) of overall sedentary time among more than 2000 children (9–10 years) and adolescents (14–15 years) in four different European countries (Denmark, Estonia, Norway and Portugal). Results demonstrated that there were different correlates across countries. The authors stated that the profiles of sedentary behaviour are culturally distinct and that interventions should target different sedentary behaviours per country. However, the authors only investigated correlates of one specific outcome (i.e. overall sedentary time). This suggests that it is also important to investigate if the correlates of television and computer time differ per country. Understanding these differences would allow future intervention developers to consider whether a standardized approach for all countries is needed or a more specific approach per country.

Therefore, the aim of this study was to examine which individual and family environmental factors are related to television and computer time separately in a large sample of 10- to 12-year-old children from Belgium, Germany, Greece, Hungary and Norway. Thus, we investigated the correlates of the two screen time behaviours separately within and across countries. We hypothesised that other correlates would be found for children’s television and computer time and for the five European countries.

## Methods

### Study protocol

Baseline cross-sectional data were used from the intervention study [17–19] as part of the ENERGY-project [20]. Intervention effects have been reported elsewhere [17, 18]. The study was conducted in five European countries (Belgium, Germany, Greece, Hungary and Norway). In total, 62 schools across the five countries agreed to participate (out of 152 invited schools; participation rate 41 %) and all children of the 5th and 6th grade (5117 eligible children) in these schools were invited to participate in the study. At baseline, 3325 children with written parental consent completed a questionnaire in the classroom. A parental questionnaire was handed out to the children and 3038 parents completed this questionnaire. Both questionnaires included items on screen time behaviours and related factors. Data collection occurred on schooldays in September and the beginning of October 2011. In total, there were 2022 child–parent dyads with complete data across the five countries (54 % girls, mean age 11.2 ± 0.8 years, mean parental age 40.5 ± 5.1 years). We compared the children in the final sample with children who were excluded from the analyses because their parents did not complete the questionnaire. Boys were more likely to be excluded and children who were excluded had higher levels of television (103.7 ± 61.1 min/day versus 97.1 ± 58.5 min/day) and computer time (81.5 ± 68.9 min/day versus 66.0 ± 59.0 min/day). The study is registered in the International Standard Randomized Controlled Trial Number Register (registration number: ISRCTN34562078; http://www.controlled-trials.com). The study protocol was approved by the ethics committee of each country. The study was approved by the Medical Ethics Committee of the University Hospital Ghent in Belgium; by the State Medical Chamber of Baden-Württemberg in Germany; by the Bioethics Committee of Harokopio University in Greece; by the Scientific and Ethics Committee of Health Sciences Council in Hungary; and by the National Committees for Research Ethics in Norway.

### Measurement of screen time behaviour

Children were asked ‘How many hours per day do you usually watch television/DVDs?’, including ten response categories: (1) Not; (2) Less than 30 min per day (recoded into 15 min per day); (3) 30 min per day; (4) 1 h per day; (5) 1.5 h per day; (6) 2 h per day; (7) 2.5 h per day, (8) 3 h per day; (9) 3.5 h per day; (10) 4 or more hours per day (recoded into 4 h per day). The question was asked separately for week- and weekend days. A similar question was asked regarding computer use: ‘How many hours per day do you usually use a computer/game console for leisure activities?’. The time using a computer/game console will be described as 'computer time' in this study. The average daily television time and computer time were calculated by following formula: ((Television/computer time on a weekday∗5) + (Television/computer time on a weekend day∗2))/7.

### Measurement of individual and family environmental factors

The child questionnaire assessed several individual factors: knowledge, attitude, preferences, self-efficacy, habit and agreement with parental rules. Both the child and parental questionnaire included items on family environmental factors. The family environmental factors were categorized into social, political, economic and physical environmental factors, in accordance with the ANGELO-framework [21] and are defined in Table 1. Two items were related to parental strictness, but the Cronbach’s alpha value was not sufficiently high (0.57) to combine them into one variable. Table 1 provides an overview of all individual and family environmental variables with the related questionnaire items and response categories. All items were asked separately for television and computer time in the questionnaire, but are combined in the table. Some individual and family environmental items were inversely recoded to attain that a higher score also represents a higher form of the variable.

**Table 1:** Questionnaire items and response categories of individual and family environmental variables

Individual variables	Item (ICC TV/PC)	Response category
Child perception of the screen time recommendations (CQ)	Children my age should not watch TV/DVD or use a computer/game console for leisure activities for more than… (ICC = 0.51/0.58)	1 = 30 min/day; 2 = 1 h/day; 3 = 2 h/day; 4 = 3 h/day; 5 = 4 h/day
Child attitude^a^ (CQ)	I think watching TV/DVD or using the computer/game console for leisure activities is good for me (ICC = 0.59/0.59)	1 = I fully agree; 2 = I partly agree; 3 = Neither agree nor disagree; 4 = I partly disagree; 5 = I fully disagree
Child preference^a^ (CQ)	I like watching TV/DVD or using a computer/game console for leisure activities (ICC = 0.56/0.51)	1 = I fully agree; 2 = I partly agree; 3 = Neither agree nor disagree; 4 = I partly disagree; 5 = I fully disagree
Child self-efficacy (CQ)	I find it hard to NOT watch TV/DVD or use a computer/game console for leisure activities (ICC = 0.57/0.69)	1 = I fully agree; 2 = I partly agree; 3 = Neither agree nor disagree; 4 = I partly disagree; 5 = I fully disagree
Child habit^a^ (CQ)	Watching TV/DVD or using a computer/game console for leisure activities is something that I do without really thinking about it (ICC = 0.60/0.63)	1 = I fully agree; 2 = I partly agree; 3 = Neither agree nor disagree; 4 = I partly disagree; 5 = I fully disagree
Child agreement with rules^a^ (CQ)	It is OK for me that my parents have rules about how much I can watch TV/DVD or use a computer/game console for leisure activities (ICC = 0.53/0.59)	1 = I fully agree; 2 = I partly agree; 3 = Neither agree nor disagree; 4 = I partly disagree; 5 = I fully disagree
Social environment: Factors regarding what is socially appropriate, acceptable or desirable as related to screen time behavioural choices		
Parental behaviour^a^ (CQ)	How often do your parents/guardians watch TV/DVD or use a computer/game console for leisure activities? (ICC = 0.73/0.46)	1 = Always; 2 = Often; 3 = Sometimes; 4 = Not often; 5 = Never
Parental co-participation (CQ)	How often do you watch TV/DVD or use a computer/game console for leisure activities with your parents/guardians? (ICC = 0.65/0.56)	1 = Never; 2 = Less than once a week; 3 = Once a week; 4 = 2–4 days a week; 5 = 5–6 days a week; 6 = Every day, once a day; 7 = Every day, more than once a day
Parental subjective norm (CQ)	If I watch TV/DVD or use a computer/game console for leisure activities, my parents/guardians think this is good for me (ICC = 0.57/0.52)	1 = I fully agree; 2 = I partly agree; 3 = Neither agree nor disagree; 4 = I partly disagree; 5 = I fully disagree
Parental perception of the screen time recommendations (PQ)	Children of same age as my child should not watch TV/DVD or use a computer/game console for leisure activities for more than… (ICC = 0.67/0.68)	1 = 30 min/day; 2 = 1 h/day; 3 = 2 h/day; 4 = 3 h/day; 5 = 4 h/day
Parental avoidance of negative modeling behaviour^a^ (PQ)	If I would like to watch TV/DVD or use a computer/game console for leisure activities, I would refrain from doing so if my child were there (ICC = 0.62/0.47)	1 = Always; 2 = Often; 3 = Sometimes; 4 = Not often; 5 = Never
Political environment: Rules and regulations regarding screen time behavioural choices		
Parental strictness item 1 (CQ)	My parents/guardians let me watch TV/DVD or use a computer/game console whenever I want (ICC = 0.73/0.67)	1 = I fully agree; 2 = I partly agree; 3 = Neither agree nor disagree; 4 = I partly disagree; 5 = I fully disagree
Parental strictness item 2 (CQ)	If I ask my parents/guardians to watch TV/DVD or use a computer/game console for leisure activities, I can do so (ICC = 0.49/0.65)	1 = I fully agree; 2 = I partly agree; 3 = Neither agree nor disagree; 4 = I partly disagree; 5 = I fully disagree
Parental rules (CQ)	My parents/guardians have rules about how much I am allowed to watch TV/DVD or use a computer/game console for leisure activities (ICC = 0.56/0.69)	1 = I fully agree; 2 = I partly agree; 3 = Neither agree nor disagree; 4 = I partly disagree; 5 = I fully disagree
Child participation in setting rules (CQ)	Did you take part in setting these rules for TV/DVD watching or using a computer/game console for leisure activities (ICC = 0.68/0.68)	0 = No; 1 = Yes
Parental monitoring^a^ (PQ)	I pay attention to the amount of time my child watches TV/DVD or uses a computer/game console for leisure activities (ICC = 0.67/0.74)	1 = Always; 2 = Often; 3 = Sometimes; 4 = Not often; 5 = Never
Parental negotiation^a^ (PQ)	I discuss the amount of time my child is allowed to watch TV/DVD or to use a computer/game console for leisure activities with him/her (ICC = 0.71/0.71)	1 = Always; 2 = Often; 3 = Sometimes; 4 = Not often; 5 = Never
Economic environment: Factors related to the ‘affordability’ of screen time behavioural choices, i.e. financial opportunities regarding, and the costs of, screen time behavioural choices		
Parental education (PQ)	What is the highest level of education you have completed?	1 = Elementary school; 2 = Secondary school; 3 = College/University (Bachelor degree or equivalent); 4 = College/University (Master degree; equivalent or more)
		Response category 1 and 2 were combined into 1 = Low education; Response category 3 and 4 were combined into 2 = High education
Physical environment: Availability and accessibility of screen time behavioural choices		
Number of TV’s in the household (CQ)	How many TV’s are there in your home? (ICC = 0.87)	1 = None; 2 = 1; 3 = 2; 4 = 3; 5 = 4; 6 = More than 4
Having a TV in the bedroom (CQ)	Do you have a TV in your own bedroom? (ICC = 0.87)	0 = No; 1 = Yes
Having a TV in the kitchen (PQ)	Do you have a TV in your kitchen? (ICC = 0.88)	0 = No; 1 = Yes
Number of computers in the household (CQ)	How many computers are there in your home? (Include laptops and iPads) (ICC = 0.92)	1 = None; 2 = 1; 3 = 2; 4 = 3; 5 = 4; 6 = More than 4
Having an own computer (CQ)	Do you have your own computer? (ICC = 0.96)	0 = No; 1 = Yes
Number of consoles in the household (CQ)	How many game consoles (e.g. Playstation, Xbox, Nintendo (Wii, GameCube, DS)) are there in your home? (ICC = 0.80)	1 = None; 2 = 1; 3 = 2; 4 = 3; 5 = 4; 6 = More than 4
Having an own console (CQ)	Do you have your own game console (e.g. Playstation, Xbox, Nintendo (Wii, GameCube, DS))? (ICC = 0.88)	0 = No; 1 = Yes

^a^These items were inversely recoded to attain that a higher score also represents a higher form of the variable; *CQ* child questionnaire, *PQ* parental questionnaire

### Reliability and validity of measures

A test-retest reliability study on the child and parental questionnaire was conducted in six schools (one school in Belgium, one school in Norway and four schools in Hungary) including 143 children and 105 parents. For screen time behaviours, good reliability was found for television time on a weekday (ICC = 0.77) and weekend day (ICC = 0.74) and for computer time on a weekend day (ICC = 0.80). Excellent reliability was found for computer time on a weekday (ICC = 0.84). For the individual and family environmental variables, results showed moderate to good reliability (ICC-values between 0.46 and 0.96). The ICC-values per item (except for parental education) are presented in Table 1 [22]. In addition, the majority of the individual and family environmental variables related to television time were used in the child and parental questionnaire of the ENERGY cross-sectional study and were generally found to have good test-retest reliability and moderate construct validity [23, 24]. Children’s television time showed a moderate to good construct validity (week ICC = 0.63; weekend ICC = 0.56), and children’s computer time showed a good construct validity for the weekend (ICC = 0.65). Only computer time on a weekday had a weak construct validity (ICC = 0.35) [23].

### Statistical analyses

SPSS 22.0 (SPSS Inc, Chicago, IL) was used to describe sample characteristics and to examine differences in demographics and screen time behaviour per country. To examine the association between individual and family environmental factors and television/computer time in each country, multilevel regression analyses were performed using MLwiN 2.22 (Centre for Multilevel Modelling, University of Bristol, UK). Multilevel modelling (two-level: pupil-school) was used to take clustering of children in schools into account. Computer/videogames time was log-transformed to obtain normal distribution. For each country, bivariate associations were examined first between each individual and family environmental factor (i.e. the independent variable) and television or computer time (i.e. the dependent variable) without adjusting for any variables. The results of the bivariate associations are reported in Additional file 1. The next step was that all individual and family environmental factors with a *p*-value of less than 0.05 (95 % confidence intervals (CI)) in the bivariate models were entered into a multivariate model, after checking for multicollinearity. In total, there were ten multivariate models (one model for television time and one model for computer for each of the five countries), all adjusted for children’s sex (dummy variable) and age (continuous variable). For both the bivariate as well as the multivariate results, we report the β-values, standard errors and significance levels.

## Results

### Sample characteristics

Table 2 presents the total and country-specific sample characteristics. All countries significantly differed from each other regarding children’s age, except for Belgium and Greece. Hungarian parents who completed the questionnaire were significantly younger than parents from all other countries and Belgian parents were significantly younger than German, Greek and Norwegian parents. Greek and Hungarian children reported significantly more television time than German and Norwegian children and Greek children also reported significantly more television time than Belgian children. Greek, Hungarian and Norwegian children reported significantly more computer time than Belgian and German children.

**Table 2:** Total and country-specific sample characteristics

	Child age (mean ± SD years)	Child sex (% girls)	Parental age (mean ± SD years)	Television time (mean ± SD min/day)	Computer time (mean ± SD min/day)
Total sample (*n* = 2022)	11.2 ± 0.8	54	40.5 ± 5.1	97.1 ± 58.5	66.0 ± 59.0
Belgium (*n* = 468)	11.1 ± 0.7	55	40.2 ± 4.4	92.7 ± 54.0	52.3 ± 49.5
Germany (*n* = 304)	10.8 ± 0.6	46	41.6 ± 5.3	87.4 ± 55.7	57.0 ± 56.1
Greece (*n* = 498)	11.5 ± 0.7	57	41.3 ± 5.2	107.3 ± 58.2	68.5 ± 57.0
Hungary (*n* = 471)	11.8 ± 0.5	54	38.8 ± 5.1	100.9 ± 65.6	77.3 ± 66.8
Norway (*n* = 301)	11.8 ± 0.5	54	41.2 ± 5.3	90.2 ± 53.2	73.7 ± 60.0

*SD* standard deviation, *min* minutes

### Individual and environmental correlates of television and computer time

Table 3 provides an overview of the results of the multivariate model for screen time behaviour by country, including β-values, standard errors and significance levels.

**Table 3:** Association between individual/family environmental variables and television or computer time in five European countries (multivariate model)

	BELGIUM		GERMANY		GREECE		HUNGARY		NORWAY	
	Television	Computer	Television	Computer	Television	Computer	Television	Computer	Television	Computer
	Β, SE (95 % CI)	Β, SE (95 % CI)	Β, SE (95 % CI)	Β, SE (95 % CI)	Β, SE (95 % CI)	Β, SE (95 % CI)	Β, SE (95 % CI)	Β, SE (95 % CI)	Β, SE (95 % CI)	Β, SE (95 % CI)
Individual variables										
Perception of recommendations	22.28, 1.93	0.10, 0.01	16.31, 2.33	0.12, 0.02	14.38, 1.98	0.08, 0.02	25.35, 2.26	0.12, 0.01	18.05, 2.41	0.10, 0.02
	(18.51;26.06)	(0.07;0.12)	(11.58;20.69)	(0.07;0.16)	(10.50;18.25)	(0.05;0.11)	(20.92;29.78)	(0.09;0.15)	(13.34;22.77)	(0.07;0.13)
Attitude	NS	0.04, 0.01	NS	NS	NS	NS	NS	NS	NS	NS
		(0.01;0.06)								
Preference	NS	0.07, 0.02	10.58, 2.78	0.09, 0.02	9.64, 2.68	0.07, 0.02	NS	0.04, 0.01	9.39, 2.99	0.09, 0.02
		(0.04;0.10)	(5.14;16.02)	(0.05;0.13)	(5.82;14.99)	(0.04;0.10)		(0.01;0.07)	(3.53;15.26)	(0.05;0.12)
Self-efficacy	NS	−0.04, 0.01	−5.05, 1.91	NS	−6.21, 1.76	−0.07, 0.01	−5.52, 1.54	−0.03, 0.01	−9.63, 1.98	−0.03, 0.01
		(−0.06; −0.01)	(−8.78; −1.31)		(−9.66; −2.76)	(−0.09; −0.04)	(−8.54; −2.50)	(−0.05; −0.01)	(−13.50; −5.76)	(−0.06;0.00)
Habit	NS	0.03, 0.01	NS	0.05, 0.02	NS	NS	NS	0.06, 0.01	NS	0.06, 0.01
		(0.00;0.05)		(0.01;0.08)				(0.03;0.08)		(0.04;0.09)
Agreement with rules	NS	–	NS	NS	NS	−0.02, 0.01	NS	NS	NS	NS
						(−0.05;0.00)				
Social environmental variables										
Parental behaviour	NS	–	NS	NS	NS	NS	7.96, 3.15	NS	9.61, 3.83	NS
							(1.78;4.14)		(2.11;17.11)	
Parental co-participation	4.07, 1.38	NS	4.77, 1.75	NS	NS	0.02, 0.01	5.47, 1.52	0.04, 0.01	5.23, 2.02	NS
	(1.37;6.78)		(1.33;8.20)			(0.00;0.04)	(2.48;8.45)	(0.02;0.05)	(1.27;9.19)	
Parental subjective norm	NS	NS	NS	NS	–	NS	−5.04, 1.93	NS	NS	NS
							(−8.83; −1.26)			
Parental perception of recommendations	NS	0.13, 0.02	18.74, 3.47	NS	–	0.08, 0.02	8.59, 2.58	0.06, 0.02	9.98, 3.42	0.08, 0.03
		(0.06;0.16)	(11.95;25.53)			(0.04;0.12)	(3.50;13.60)	(0.01;0.10)	(3.28;16.68)	(0.03;0.13)
Parental avoidance of negative modeling behaviour	–	–	NS	−0.03, 0.02	–	–	–	−0.02, 0.01	–	–
				(−0.07;0.00)				(−0.04;0.00)		
Political environmental variables										
Parental strictness 1	−5.16, 1.46	NS	NS	NS	NS	−0.03, 0.01	NS	NS	NS	NS
	(−8.02; −2.30)					(−0.06; −0.01)				
Parental strictness 2	NS	NS	NS	−0.07, 0.03	−9.64, 2.68	NS	NS	−0.05, 0.02	NS	NS
				(−0.13; −0.02)	(−14.90; −4.39)			(−0.08; −0.01)		
Parental rules	NS	–	5.41, 1.62	NS	4.72, 1.65	–	3.47, 1.52	NS	–	–
			(2.23;8.58)		(1.50;7.95)		(0.48;6.45)			
Child participation in setting rules	–	–	–	–	NS	–	NS	–	–	–
Parental monitoring	−8.04, 2.18	−0.04, 0.02	NS	–	NS	–	NS	NS	NS	–
	(−12.31; −3.77)	(−0.07; −0.01)								
Parental negotiation	NS	NS	–	–	−6.57, 2.32	NS	NS	–	NS	−0.04, 0.02
					(−11.12; −2.01)					(−0.08; −0.01)
Economic environmental variables										
Parental education	NS	NS	–	–	–	NS	NS	NS	–	–
Physical environmental variables										
TV										
Number of TV’s in the household	NS	n/a	NS	n/a	NS	n/a	NS	n/a	NS	n/a
Having a TV in the bedroom	NS	n/a	NS	n/a	NS	n/a	NS	n/a	NS	n/a
Having a TV in the kitchen	–	n/a	–	n/a	–	n/a	–	n/a	–	n/a
Computer										
Number of computers in the household	n/a	–	n/a	NS	n/a	–	n/a	NS	n/a	NS
Having an own computer	n/a	NS	n/a	NS	n/a	–	n/a	0.10, 0.03	n/a	NS
								(0.04;0.16)		
Number of consoles in the household	n/a	0.03, 0.01	n/a	NS	n/a	NS	n/a	NS	n/a	–
		(0.00;0.04)								
Having an own console	n/a	–	n/a	NS	n/a	NS	n/a	NS	n/a	NS

Multivariate model, adjusted for age and sex; *SE* standard error, *95* % *CI* 95 % confidence interval, *NS* significant in univariate model, but not in multivariate model, − not significant in univariate model and therefore not included in multivariate model, *n*/*a* not applicable

#### Individual variables

In all countries, children’s perception of the recommendations for television and computer time was significantly associated with both screen time behaviours: the more hours children thought it was recommended to watch television and use a computer, the more time they engaged in watching television and using the computer. Children’s higher preference for television watching and/or computer use and lower self-efficacy to control television watching and/or computer use were significantly associated with more television and/or computer time in all countries. Higher habit strength regarding computer use was significantly associated with more computer use. A more positive attitude towards computer use was associated with more computer time in Belgium. A higher child agreement with rules on computer use was significantly associated with less computer time in Greece.

#### Social environmental variables

In all countries, parental perception of the recommendations for children’s television and computer time was significantly associated with either one or both screen time behaviours: the more hours parents thought it was recommended for children to watch television or use a computer, the more time their child engaged in watching television and/or using the computer. Higher levels of parental co-participation in television watching was significantly associated with more television time for children from all countries, except Greece. Higher levels of parental television time was only significantly associated with more television time for children from Hungary and Greece and a higher parental subjective norm regarding television time was significantly associated with less television time for children from Hungary. A higher score on parental avoidance of negative computer modeling behaviour was significantly associated with less computer time for children from Germany and Hungary.

#### Political environmental variables

Having rules regarding television time was significantly inversely associated with children’s television time in all countries, except for Belgium and Norway. More parental monitoring of children’s television and computer time was only significantly associated with less television and computer time for children from Belgium.

#### Economic environmental variables

Parental education was not significantly associated with television or computer time in all countries.

#### Physical environmental variables

There were no significant associations between any of the physical environmental variables and children’s television time in all countries. The number of game consoles in the household was significantly positively associated with children’s computer time in Belgium and having an own computer was significantly associated with more computer time in Hungary.

## Discussion

The present study investigated which individual and family environmental factors are related to television and computer time in 10- to 12-year-old European children. In general, more evidence was found for individual and social environmental factors than for political and physical environmental factors. This suggests that interventions aiming to reduce screen time could focus on changing children’s individual knowledge, beliefs and habits as well parental social factors. Children’s perception of the screen time recommendations, preferences and self-efficacy were all related to one or both screen time activities. Similar results were found for parental perception of screen time recommendations and parental co-participation. An important finding was that both children’s and parental perceptions of the recommendations for television and computer time were positively associated with children’s television and computer time. Increasing knowledge on recommendations or health consequences is generally not likely to induce large behavioural changes, but providing information might be a necessary first step in intervention programmes to increase awareness about potentially unhealthy behaviours [24]. This could especially be the case for screen time behaviour as the majority of people are not aware of the recommendation for children to spend no more than 2 h per day in front of a screen [25, 26]. A generic media campaign among the different European countries could therefore acquaint people with the screen time recommendations. Providing information to European children and parents on the screen time recommendations might possibly lead to a reduction in children’s screen time behaviour.

Although many significant bivariate associations with television and computer time were found, only a few remained significant in the final multivariate model. For example, no physical environmental variables were associated with children’s television time across all countries in the final model, although the number of televisions in the household and having a television in the bedroom have previously been identified as important correlates of sedentary behaviour in this particular age group [12, 13]. This could imply that the influence of the physical environment attenuates when individual or other environmental variables are considered. Moreover, parental education was not associated with both screen time behaviours in the multivariate model, suggesting that the differences in sedentary behaviour according to parental education might possibly be due to differences in the social or political environment. Future mediation analyses could further explore this hypothesis by investigating if the inverse relationship between parental education and screen time behaviour is mediated by social or political environmental variables. A recent study already confirmed that parental television time is important in explaining parental educational differences in children’s television time [27], but future studies could also investigate other social or political environmental variables as potential mediating factors, such as parental co-participation and parental rules.

Besides the similarities in correlates across screen time behaviours and countries, this study also identified different correlates for television and computer time and for the five European countries. Slightly more individual variables were related to children’s computer time (e.g. preferences and habit), while more social environmental variables were associated with children’s television time. This could be explained by the fact that activities on the computer or game console are often performed individually without parents, whereas watching television is more likely to be a family activity [28]. An intervention focusing on the reduction of screen time could therefore focus on changing different related factors for television and computer time and might employ intervention strategies according to the specific activity. A possible strategy to reduce children’s computer time could be to focus on changing individual factors, for example by using cues and prompts as a technique to change children’s computer habits (i.e. to help reminding children to limit their computer time). On the other hand, a specific strategy to reduce television time could focus on changing the parental social environment, for example by informing parents on fun alternatives to do as a family or even by organising such family activities. It appears that even when children grow older, watching television together remains an important family activity [29], so targeting this factor already in childhood could be important. As the present study investigated only two screen time behaviours, future studies could also investigate differences in correlates of other emerging screen time behaviours (e.g. smartphone use) or other sedentary behaviours (e.g. passive transportation).

There were also different correlates of screen time behaviour across the European countries. For example, all social environmental variables were related to television time among Hungarian children, whereas no social environmental variables were associated with television time among Greek children. In Greece, the results suggest that it may be more important to target the more political environmental factors –i.e. rules and regulation related family environmental factors such as parental strictness, having parental rules, and parental negotiation- instead of the social environmental factors to reduce children’s television time. It is, however, difficult to explain this difference. It could be that that Greek parents have in general a more authoritative parenting style, characterized by more strictness and control on their children’s television viewing [30].

An implication, based on the different correlates found in the present study, is that developing a completely standardized intervention to reduce general screen time for several European countries might be less effective. It is possible that the intervention would focus on changing factors that are not associated with a particular screen time behaviour or with screen time behaviour in a particular country. This is in accordance with the study findings of Babey and colleagues [15] who stated that understanding the differences in correlates of different screen time behaviours can inform more effective interventions and with the study findings of van Sluijs and colleagues [16] who advocated that a single strategy to reduce sedentary behaviour is less likely to be effective across Europe. The reason why differences in correlates exist between countries might be the specific characteristics of a country, such as the cultural attitude towards screen time behaviour or the typical family structure or context that could influence individual and family environmental factors [31]. It has also been advocated that children from European countries where parents have a more permissive parenting style engage in more individual television viewing, whereas children from countries where parents have a less permissive parenting style watch more television in the presence of other family members [32]. Furthermore, it might be that the neighbourhood environment differs across European countries, which could also influence children screen time. For example, the presence of sidewalks and parks has been found to be inversely associated with children’s screen time [33]. It can thus be concluded that the differences found across countries is a complex matter that needs further investigation. Nevertheless, there are also many similarities between both screen time behaviours and especially between countries, suggesting that cross-European intervention strategies to reduce both computer and television time are possible if small adaptations can be made or if different emphases can be laid per screen time behaviour or per country.

The current study has some limitations that are further discussed below. First, using cross-sectional data rules out the possibility to draw conclusions about causality. A second limitation is the possibility of social desirable responses because of the use of self-reported child and parental data. Although the individual and family environmental variables mostly had good (ICC >0.60 in more than 60 % of the variables) or moderate (ICC >0.40) test-retest reliability, psychometric properties could be improved. Also, the item to assess computer time on weekdays showed weak construct validity. Despite these shortcomings that are inherent to questionnaires, they are common and often the only possible method in studies to assess screen time behaviours because of their low cost and participant burden [34]. Objective measurements such as accelerometers would not be useful for the present study, because they only measure the total sitting time and do not have the ability to assess specific sedentary activities, such as television and computer time. Another method that can assess screen time behaviours and that overcomes some of the problems associated with questionnaires such as recall bias, are diaries and ecological momentary assessment methods [34]. However, compliance with such methods may be low because of the high participant burden which could especially be challenging in large cross-European studies such as the ENERGY-project. Another limitation is that the data were collected 4 years ago. Since screen-based behaviours evolve rapidly because of new technologies, it might be that some correlates have evolved too and further research is warranted to assess correlates and potential determinants of other screen-based activities, for example related to tablet and mobile smart phone use. Finally, there are some study aspects that limit the generalisability of the findings: although there was a relatively large total sample of children, the sample of children per country was not a random and thus representative sample. Moreover, the findings are restricted to children aged 10 to 12 years. Finally, children who were excluded from the analyses because their parents did not complete the questionnaire were more likely to be boys and had higher levels of both television and computer time. Important strengths are the investigation of correlates of two screen time activities in a large sample of children across five countries separately, and the inclusion of a broad range of individual and family environmental factors. Future studies could also consider the influence of other environmental factors, such as the school, neighbourhood or even the grand-parental environment.

## Conclusion

Children’s and parents’ perception of the screen time recommendations were related to children’s television and computer time in all countries, suggesting that a generic European intervention focusing on increasing the knowledge and awareness regarding screen time behaviour could be relevant. Most evidence was found for an association between screen time behaviour and individual and social environmental factors among 10- to 12-year old European children, although somewhat more individual factors were related to children’s computer time and more parental social environmental factors to children’s television time. It could thus be important to take the different correlates of television and computer time into account when developing an intervention to reduce screen time. Although most correlates of screen time behaviour were similar across countries, cross-European interventions could lay different emphases per country in order to match the identified correlates.

## Additional file

Additional file 1:
**Bivariate associations between individual and family environmental factors and television or computer time per country.** (DOCX 25 kb)

## Abbreviations

ICC: Intraclass correlation coefficient
