# Supplementary material for: Individual and family environmental correlates of television and computer time in 10- to 12-year-old European children: the ENERGY-project
Source: BMC Public Health. 2015 Sep 18;15:912. doi: 10.1186/s12889-015-2276-2 (PMC4574577; doi:10.1186/s12889-015-2276-2)
Supplement: Additional file 1: — Bivariate associations between individual and family environmental factors and television or computer time per country. (DOCX 25 kb) [file 12889_2015_2276_MOESM1_ESM.docx]

Additional file 1: Bivariate associations between individual and family environmental factors and television or computer time in five European countries

|  | **BELGIUM** | | **GERMANY** | | **GREECE** | | **HUNGARY** | | **NORWAY** | | |
| --- | --- | --- | --- | --- | --- | --- | --- | --- | --- | --- | --- |
|  | *Television*  *Β, SE (95%CI)* | *Computer*  *Β, SE (95%CI)* | *Television*  *Β, SE (95%CI)* | *Computer*  *Β, SE (95%CI)* | *Television*  *Β, SE (95%CI)* | *Computer*  *Β, SE (95%CI)* | *Television*  *Β, SE (95%CI)* | *Computer*  *Β, SE (95%CI)* | *Television*  *Β, SE (95%CI)* | | *Computer*  *Β, SE (95%CI)* |
| **Individual variables** | | | | | | | | | | | |
| Perception of recommendations | **-24.50, 1.96**  **(20.66;28.35)** | **0.15, 0.02**  **(0.12;0.18)** | **24.12, 2.69**  **(18.84;29.40)** | **0.22, 0.02**  **(0.18;0.27)** | **19.35, 2.11**  **(15.22; 23.48)** | **0.15, 0.02**  **(0.11;0.18)** | **33.31, 2.27**  **(28.85;37.76)** | **0.19, 0.02**  **(0.16;0.22)** | **22.80, 2.63**  **(17.64;27.97)** | | **0.18, 0.02**  **(0.15;0.22)** |
| Attitude | **-6.97, 1.96**  **(-10.81;-3.13)** | **-0.11, 0.01**  **(-0.14;-0.08)** | **-11.05, 2.71**  **(-16.35;-5.74)** | **-0.13, 0.02**  **(-0.18;-0.09)** | **-9.71, 2.32**  **(-14.26; -5.17)** | **-0.11, 0.02**  **(-0.14;-0.08)** | **-12.81, 2.17**  **(-17.07;-8.55)** | **-0.13, 0.01**  **(-0.15;-0.10)** | **-13.82, 2.87**  **(-19.45;-8.19)** | | **-0.12, 0.02**  **(-0.16;-0.08)** |
| Preference | **-14.48, 2.92**  **(-20.20;-8.75)** | **-0.15, 0.02**  **(-0.18;-0.12)** | **-21.12, 3.06**  **(-27.12;-15.13)** | **-0.19, 0.02**  **(-0.23;-0.16)** | **-16.00, 2.43**  **(-20.75;-11.24)** | **-0.14, 0.02**  **(-0.17;-0.11)** | **-19.40, 2.83**  **(-24.95;-13.85)** | **-0.15, 0.01**  **(-0.18;-0.12)** | **-19.17, 3.24**  **(-25.52;-12.82)** | | **-0.20, 0.02**  **(-0.23;-0.16)** |
| Self-efficacy | **-6.57, 1.74**  **(-9.99;-3.15)** | **-0.10, 0.01**  **(-0.12;-0.07)** | **-11.39, 2.26**  **(-15.82;-6.97)** | **-0.13, 0.02**  **(-0.16;-0.09)** | **-11.74, 1.84**  **(-15.34;-8.15)** | **-0.11, 0.01**  **(-0.13;-0.09)** | **-10.59, 1.86**  **(-14.23; -6.95)** | **-0.10, 0.01**  **(-0.12;-0.08)** | **-14.93, 2.21**  **(-19.26;-10.60)** | | **-0.11, 0.02**  **(-0.14; -0.08)** |
| Habit | **-7.52, 1.87**  **(-11.18;-3.86)** | **-0.09, 0.01**  **(-0.12;-0.06)** | **-12.81, 2.39**  **(-17.49;-8.12)** | **-0.12, 0.02**  **(-0.16;-0.08)** | **-10.16, 1.78**  **(-13.65;-6.66)** | **-0.05, 0.01**  **(-0.08;-0.03)** | **-10.68, 1.91**  **(-14.42; -6.94)** | **-0.13, 0.01**  **(-0.16; -0.11)** | **-9.67, 2.43**  **(-14.44;-4.90)** | | **-0.13, 0.02**  **(-0.16;-0.10)** |
| Agreement with rules | **5.52, 1.88**  **(1.83;9.21)** | 0.01, 0.01  (-0.02;0.03) | **11.21, 2.56**  **(6.19;16.24)** | **0.07, 0.02**  **(0.03;0.10)** | **9.13, 2.09**  **(5.04; 13.22)** | **0.04, 0.02**  **(0.01;0.07)** | **5.64, 2.14**  **(1.45; 9.82)** | **0.06, 0.02**  **(0.03, 0.10)** | **7.68, 2.57**  **(2.64; 12.72)** | | **0.07, 0.02**  **(0.03;0.11)** |
| **Social environmental variables** | | | | | | | | | | | |
| Parental behaviour | **-7.85, 3.23**  **(-14.19; -1.51)** | -0.04, 0.02  (-0.07;0.00) | **-7.70, 3.93**  **(-15.40;-0.01)** | **-0.07, 0.02**  **(-0.12;-0.02)** | **-5.45, 2.09**  **(-9.54;-1.36)** | **-0.04, 0.02**  **(-0.07;-0.01)** | **-22.43, 3.55**  **(-29.40;-15.46)** | **-0.09, 0.02**  **(-0.13;-0.06)** | **-17.21, 4.64**  **(-26.30;-8.13)** | **-0.06, 0.02**  **(-0.10;-0.01)** | |
| Parental co-participation | **8.21, 1.58**  **(5.11; 11.31)** | **0.04, 0.01**  **(0.01;0.06)** | **12.61, 2.10**  **(8.49;16.72)** | **0.05, 0.02**  **(0.01;0.08)** | **3.61, 1.68**  **(0.31;6.90)** | **0.05, 0.01**  **(0.03;0.07)** | **12.82, 1.72**  **(9.45;16.19)** | **0.07, 0.01**  **(0.05;0.09)** | **11.20, 2.34**  **(6.61;15.78)** | **0.06, 0.02**  **(0.02;0.10)** | |
| Parental subjective norm | **-8.12, 2.44**  **(-12.92; -3.32)** | **-0.06, 0.02**  **(-0.09;-0.02)** | **-12.49, 3.22**  **(-18.81;-6.17)** | **-0.07, 0.03**  **(-0.13;-0.02)** | -3.01, 2.35  (-7.63;1.60) | **-0.08, 0.02**  **(-0.11;-0.04)** | **-13.11, 2.31**  **(-17.63;-8.58)** | **-0.10, 0.02**  **(-0.13;-0.06)** | **-8.48, 3.34**  **(-15.02;-1.94)** | **-0.05, 0.02**  **(-0.10;-0.01)** | |
| Parental perception of recommendations | **-16.05, 2.93**  **(10.32; 21.79)** | **0.20, 0.03**  **(0.15;0.25)** | **30.13, 3.99**  **(22.31;37.95)** | **0.11, 0.04**  **(0.04;0.19)** | 5.64, 3.49  (-1.21;12.48) | **0.16, 0.03**  **(0.11;0.21)** | **16.90, 3.19**  **(10.65;23.15)** | **0.16, 0.03**  **(0.11; 0.22)** | **13.20, 4.08**  **(5.21;21.18)** | **0.21, 0.03**  **(0.14;0.27)** | |
| Parental avoidance of negative modeling behaviour | 3.74, 2.04  (-0.25;7.73) | -0.01, 0.01  (-0.04;0.02) | **10.81, 2.91**  **(5.10;16.52)** | **0.07, 0.02**  **(0.03;0.12)** | 1.28, 2.27  (-3.16;5.72) | -0.02, 0.01  (-0.05;0.00) | 3.43, 2.49  (-1.46;8.32) | **0.04, 0.02**  **(0.01, 0.06)** | 0.94, 3.90  (-6.71;8.58) | 0.00, 0.02  (-0.04;0.04) | |
| **Political environmental variables** | | | | | | | | | | | |
| Parental strictness 1 | **-8.35, 1.65**  **(-11.58;-5.11)** | **-0.04, 0.01**  **(-0.06;-0.01)** | **-11.44, 2.38**  **(-16.10;-6.79)** | **-0.11, 0.02**  **(-0.15;-0.06)** | **-8.60, 1.89**  **(-12.30;-4.90)** | **-0.09, 0.01**  **(-0.11;-0.06)** | **-10.38, 1.86**  **(-14.03;-6.74)** | **-0.07, 0.01**  **(-0.10;-0.05)** | **-8.67, 2.44**  **(-13.46;-3.89)** | | **-0.04, 0.02**  **(-0.07;0.00)** |
| Parental strictness 2 | **-14.89, 3.08**  **(-20.91; -8.86)** | **-0.11, 0.02**  **(-0.15;-0.06)** | **-19.19, 4.04**  **(-27.11;-11.28)** | **-0.20, 0.03**  **(-0.26;-0.14)** | **-18.76, 2.89**  **(-24.42;-13.11)** | **-0.11, 0.02**  **(-0.14;-0.07)** | **-9.24, 2.94**  **(-14.99;-3.48)** | **-0.11, 0.02**  **(-0.16;-0.07)** | **-13.09, 4.10**  **(-21.13;-5.06)** | | **-0.09, 0.03**  **(-0.15;-0.02)** |
| Rules | **4.27, 1.65**  **(1.04; 7.50)** | -0.01, 0.01  (-0.04, 0.01) | **6.44, 2.10**  **(2.32;10.57)** | **0.04, 0.02**  **(0.00;0.07)** | **8.77, 1.84**  **(5.17;12.38)** | 0.02, 0.01  (0.00;0.05) | **7.65, 1.92**  **(3.88;11.41)** | **0.04, 0.02**  **(0.01;0.07)** | 2.95, 2.20  (-1.36;7.26) | | 0.00, 0.02  (-0.03;0.03) |
| Child participation in setting rules | 4.46, 2.99  (-1.40;10.32) | -0.01, 0.01  (-0.05;0.04) | 6.00, 3.19  (-0.25;12.26) | 0.04, 0.03  (-0.03;0.11) | **10.15, 3.24**  **(3.80;16.50)** | **0.04, 0.02**  **(-0.01;0.08)** | **8.17, 3.68**  **(0.97;15.37)** | 0.00, 0.03  (-0.05;0.05) | 1.21, 4.26  (-7.13;9.55) | | -0.03, 0.03  (-0.09;0.04) |
| Parental monitoring | **13.19, 2.45**  **(8.38;18.00)** | **0.08, 0.02**  **(0.04;0.13)** | **15.93, 4.01**  **(8.08;23.79)** | 0.06, 0.04  (-0.01;0.13) | **12.16, 3.10**  **(6.08;18.24)** | 0.05, 0.03  (0.00;0.10) | **10.38, 3.61**  **(3.31;17.44)** | **0.07, 0.02**  **(0.02;0.12)** | **15.70, 4.88**  **(6.14;25.25)** | | 0.03, 0.04  (-0.04;0.11) |
| Parental negotiation | **7.21, 2.08**  **(3.13;11.29)** | **0.04, 0.02**  **(0.01;0.07)** | 0.24, 2.80  (-5.24;5.73) | -0.01, 0.02  (-0.06;0.03) | **9.87, 2.68**  **(4.61;15.13)** | **0.05, 0.02**  **(0.00;0.09)** | **6.54, 2.66**  **(1.31;11.756)** | **0.04, 0.02**  **(-0.01;0.08)** | **-7.08, 3.41**  **(-13.76;-0.39)** | | **-0.10, 0.02**  **(-0.14;-0.05)** |
| **Economic environmental variables** | | | | | | | | | | | |
| Parental education | -**20.26, 5.12**  **(-30.30; -10.23)** | **-0.13, 0.04**  **(-0.20;-0.05)** | -5.68, 7.32  (-20.03;8.68) | -0.05, 0.06  (-0.17;0.08) | -8.26, 5.22  (-18.50;1.98) | **-0.12, 0.04**  **(-0.19;-0.05)** | **-27.04, 6.67**  **(-40.11;-13.97)** | **-0.20, 0.05**  **(-0.29;-0.10)** | -1.72, 6.29  (-14.05;10.61) | | -0.03, 0.05  (-0.12;0.07) |
| **Physical environmental variables** | | | | | | | | | | | |
| *TV* | | | | | | | | | | | |
| Number of TV’s in the household | **7.21, 2.26**  **(2.79;11.64)** | n/a | **8.10, 2.87**  **(2.49;13.72)** | n/a | **6.72, 2.91**  **(1.00;12.43)** | n/a | **10.43, 2.73**  **(5.07;15.78)** | n/a | **8.42, 2.89**  **(2.75;14.08)** | | n/a |
| Having a TV in the bedroom | **18.78, 5.62**  **(7.76;29.80)** | n/a | **27.20, 6.68**  **(14.10;40.29)** | n/a | **10.95, 5.39**  **(0.39;21.51)** | n/a | **18.28, 6.32**  **(5.89;30.67)** | n/a | **13.97, 6.61**  **(1.01;26.93)** | | n/a |
| Having a TV in the kitchen | **11.13, 6.49**  **(-1.58;23.84)** | n/a | 30.09, 24.73  (-18.37;78.56) | n/a | 10.93, 6.44  (-1.68;23.55) | n/a | 7.87, 8.60  (-8.99;24.72) | n/a | -5.38, 14.54  (-33.87;23.11) | | n/a |
| *Computer* | | | | | | | | | | | |
| Number of computers in the household | n/a | 0.03, 0.02  (0.00;0.06) | n/a | **0.05, 0.02**  **(0.00;0.09)** | n/a | 0.01, 0.02  (-0.02;0.05) | n/a | **0.05, 0.02**  **(0.01;0.08)** | n/a | | **0.04, 0.02**  **(0.00;0.07)** |
| Having an own computer | n/a | **0.14, 0.04**  **(0.06;0.22)** | n/a | **0.11, 0.05**  **(0.01;0.22)** | n/a | 0.07, 0.04  (0.00;0.15) | n/a | **0.24, 0.04**  **(0.16;0.32)** | n/a | | **0.13, 0.05**  **(0.04;0.23)** |
| Number of consoles in the household | n/a | **0.05, 0.01**  **(0.03;0.08)** | n/a | **0.07, 0.02**  **(0.03;0.10)** | n/a | **0.05, 0.01**  **(0.02;0.07)** | n/a | **0.05, 0.02**  **(0.02;0.08)** | n/a | | 0.03, 0.02  (0.00;0.06) |
| Having an own console | n/a | 0.05, 0.05  (-0.05;0.15) | n/a | **0.20, 0.07**  **(0.07;0.33)** | n/a | **0.11, 0.04**  **(0.03;0.18)** | n/a | **0.17, 0.04**  **(0.09;0.26)** | n/a | | **0.15, 0.05**  **(0.05;0.25)** |

SE, standard error; 95% CI, 95% confidence interval; n/a, not applicable; bold values indicate a significant association
